# Gender Differences in Neuromuscular, Haematological and Urinary Responses during Padel Matches

**DOI:** 10.3390/ijerph18115864

**Published:** 2021-05-29

**Authors:** Francisco Pradas, Alejandro García-Giménez, Víctor Toro-Román, Nicolae Ochiana, Carlos Castellar

**Affiliations:** 1 ENFYRED Research Group, University of Zaragoza, 22001 Huesca, Spain; franprad@unizar.es (F.P.); garciagimenezalejandro@gmail.com (A.G.-G.); castella@unizar.es (C.C.); 2 Faculty of Sports Science, University of Extremadura, University Avenue, 10003 Cáceres, Spain; 3 Department of Physical Education and Sports Performance, Faculty of Movement, Sports and Health Sciences, Vasile Alecsandri University of Bacău, 600115 Bacău, Romania; sochiana@ub.ro

**Keywords:** racket sports, jump, fatigue, hand grip strength, performance

## Abstract

Research on the acute physiological response to a padel match is limited. The present study aimed to: (a) evaluate neuromuscular, urinary, and hematological responses after simulated padel competition (SC) and (b) analyze possible gender differences. In this study, 28 high-level padel players participated (men = 13, age = 26.83 ± 6.57 years; women = 15, age = 30.07 ± 4.36 years). The following parameters were analyzed before and after SC: neuromuscular (hand grip strength, squat jump (SJ), countermovement jump (CMJ), and Abalakov jump (ABK)), hematological (red blood cells, hemoglobin, and hematocrit), and urinary (pH, specific gravity, microalbuminuria, and red blood cells). Significant gender differences were found in neuromuscular and hematological responses, with men obtaining higher values (*p* < 0.05). For the SC influence, changes were noted in ABK and microalbuminuria (*p* < 0.05). The percentages of change in hand grip strength, SJ (height and watts), CMJ (height), and ABK (height) were higher for men than women (*p* < 0.05). SC negatively influenced the neuromuscular parameters to a greater extent in women. Our results could be related to gender differences in game actions, the temporal structure, and anthropometric and physiological characteristics. Game dynamics and a different organic response between male and female padel playing were confirmed.

## 1. Introduction

Padel is an intermittent racket sport that is played in pairs on a small artificial lawn court (20 × 10 m) surrounded by glass walls and metal meshing, on which balls bounce [1]. Playing padel has become a sport preference for society today [2,3]. In recent years, padel sport practice has exponentially grown in Spain and elsewhere in the world for both genders [2]. This increase could lie in the various advantages that padel offers over other racket sports; for example, no special technical skills are required to start this sport, it can be played both indoors and outdoors, and padel materials are not expensive [2,4,5]. Interest in this sport has grown as different benefits for physical condition and body composition from regularly playing padel have been reported [6].

Regarding its game dynamics, during rallies, padel combines short, highly intensive actions (0.7–1.5 s) and other longer, less intensive actions (9–15 s), which are alternated with pauses and rests between points that normally last 10–20 s [7]. Padel is characterized by being an intermittent sport that is predominantly aerobic and one that includes short high-intense and very high-intense game actions, during which the phosphagen system prevails (ATP-PCr) [8,9,10]. Indeed, low-intensity and rest periods occupy around 60% of playing time [11]. During a padel match, players have to perform rapid movements and constantly change direction in very short times. This means that strength, agility, and speed are important physical qualities to play this sport [10]. Compared to other racket sports, padel’s characteristics have drawn more researchers’ interest in recent years [4,10,12,13].

In gender terms, previous studies report major differences between men and women in the temporal structure and for game actions while playing padel matches [13,14,15]. Tactical game actions, such as number of lobs per point or number of smashes per point, are more numerous for women than men [16]. Women padel players also more frequently use forehand services and backhand services than their male counterparts, and they perform fewer backhand volleys than men [17]. Men perform a higher percentage of winning smashes than women [13]. For the temporal structure, the total time, playing time, and pieces of play per match are longer, and there are more pieces of play per match in a female padel player than a male padel player, in which short and more intense actions predominate [17,18]. Thus, according to match characteristics per gender, both the load and physiological responses can differ.

Racket sports seem to lead to relevant acute responses on the organism [19,20]. Some authors have previously reported more delayed onset muscle soreness (DOMS) and reduced maximum voluntary contractions (MVC) after several tennis matches [19]. In simulated badminton matches, changes in hemoglobin and hematocrit levels have been found [20]. In other racket sports, alterations to neuromuscular function have also been noted after playing matches [21,22]. Yet, despite the marked increase in the number of scientific publications on padel, today’s scientific literature regarding an acute physiological response after a padel match is still limited and unknown [10], and no research works can be found that have analyzed neuromuscular responses in both genders after a padel match. As with other racket sports, a padel match is believed to possibly modify players’ neuromuscular responses. Knowledge of acute neuromuscular and physiological response after a padel match could prove to be a key parameter to control and program future training loads. Therefore, the objectives of this study were to analyze (a) neuromuscular, urinary, and hematological responses after a simulated padel competition (SC) and (b) possible gender differences.

## 2. Materials and Methods

### 2.1. Participants

In the present study, 28 high-level padel players participated (men = 13; women = 15), who had all participated in the professional World Padel Tour (WPT) circuit in the past 7 years. The sample was selected by convenience due to the difficulty of finding these types of athletes. All the evaluations were performed after the last day of the WPT. The sample’s characteristics are shown per gender in Table 1.

All the participants were informed about the aim of the study and gave their informed consent. A code was assigned to each participant to collect and process samples to maintain their anonymity. This research work was carried out according to the Declaration of Helsinki ethic guidelines, updated at the World Medical Assembly in Fortaleza (Brazil) in 2013 for research with human subjects. The Clinical Research Ethics Committee of the Department of Health and Consumption of the Government of Aragón (Spain) approved the research project (code: 21/2012).

The participants had to meet the following criteria to be included in this study: having played in the WPT in at least the last 5 years, finishing SC, not following any special diet, not being on specific medication or over-the-counter medication, and not having any injuries or illness during the research or at least 5 months before the study began.

### 2.2. Procedures

Given the difficulties of analyzing acute neuromuscular and physiological parameters during WPT matches, SC was designed. The SC characteristics appear in Table 2. SC consisted of organizing a padel match to reproduce a competitive situation similar to an official one and in line with International Padel Federation rules [23]. Matches were played on an open-air court. Players’ training intensity and volume were reduced in the 2 days before SC to reduce fatigue.

SC was organized in accordance with the official regulations for professional tournaments, and all the matches were played with the best of three sets [23]. Matches ended in a tie break if there was a tie after six games. Before starting SC, players did a standard 15-min warm up divided into a 5-min movement and general warm up session and a 10-min specific technical warm up on the court. Players were monitored during SC by pulsometers (Vantage M, Polar, Finland). During matches, what the players drank was controlled. Drinks consisted of bottled mineral water. Players could drink ad libitum during matches.

The total time was the full match time, from the time it began to the time it ended and included game and rest periods. Real time was the time from when a point began (when the serving player hit the ball) until the end. Rest time was from the end of one point to the beginning of the next point [10].

### 2.3. Anthropometric Measurements

The participants’ morphological characteristics were evaluated in the morning and always under the same conditions. Body height was measured to the nearest 0.1 cm, using a wall-mounted stadiometer (Seca 220, Hamburg, Germany). Body weight was measured to the nearest 0.01 kg on calibrated electronic digital scales (Seca 769, Hamburg, Germany) in the nude and barefoot. A Holtain© 610ND (Holtain, Crymych, UK) skinfold compass, accurate to ±0.2 mm, was employed for the anthropometric assessments. These measurements were height, weight, and six skinfolds (abdominal, suprailiac, subscapular, tricipital, thigh, leg). Yushaz equations were used to calculate the fat percentage [24]. All the measurements were taken by the same operator, who was skilled in kinanthropometric techniques, in accordance with the International Society for the Advancement of Kinanthropometry recommendations [25]. Body weight was measured both pre-SC and post-SC.

### 2.4. Testing Protocol

One week before SC, a maximum progressive test was run in the laboratory on a treadmill (Pulsar HP, Cosmos, Nussdorf, Germany) to determine the physical performance parameters. This test was performed at a 1% slope, starting at a speed of 8 km·h^−1^ and incorporating 1 km·h^−1^ increments every minute. Before strength testing began, the participants warmed up on a treadmill at the speed of 6 km·h^−1^ for 5 min. Gases were analyzed by an Oxycon Pro analyzer (Jaegger, Germany). A pulsometer (Vantage M, Polar, Finland) was used to evaluate the maximum heart rate.

### 2.5. Neuromuscular Test

Neuromuscular function was evaluated by hand grip strength, the squat jump (SJ), the countermovement jump (CMJ), and the Abalakov jump (ABK) [26,27]. Assessments were carried out 1 h before and after SC (2–5 min after). The jump tests were selected to measure the neuromuscular function of leg extensor muscles because they can achieve this at a high reliability level [28,29]. A jump mat system (Chronojump Boscosystems, Barcelona, Spain) was employed to measure jump heights, watts, and times. Three attempts were allowed for all jumps, with a 30 s rest period between jumps. The best jump was chosen to be later analyzed. All the jump tests were carried out in line with the guidelines of both Markovic et al. [30] and Rodríguez-Rosell et al. [28].

Hand grip strength was measured by a Takei 5101 dynamometer (Takei Instruments Ltd., Tokyo, Japan). The participants did two MVCs while fully extending hands and arms. Both the dominant and non-dominant arms were assessed. The dynamometer gripping piece was adapted to the participants’ hands [31]. They had two attempts, and the mean value was selected to be later analyzed.

### 2.6. Blood Samples and Analyses

Two venous blood samples were taken (5 mL) from the antecubital vein from each participant. Blood samples were collected in coded Vacutainer tubes containing ethylenediaminetetraacetic acid (EDTA) as an anticoagulant. The first sample was taken 120 min before SC after a minimum period of approximately 8 h since the last eaten meal. The second sample was taken when matches ended (7–10 min after). After the first blood sample, the participants ingested a similar breakfast, which consisted of a bottle of drink with 5% glucose solution.

Hematological parameters (red blood cells, hematocrit, hemoglobin) were determined with an analyzer model Coulter model AcT diff in the laboratory of the San Jorge University Hospital (Huesca, Spain).

### 2.7. Urine Samples and Analyses

The first urine sample in the morning and the first urine sample after SC were taken from all the subjects. They were collected in polyethylene tubes previously washed with diluted nitric acid and frozen at −80 °C until analyzed, and once the container was handed over, it was measured and codified. Prior to analyses, samples were thawed and homogenized by shaking.

A 10 mL quantity was used to obtain the different evaluated parameters. Specific gravity was analyzed in situ with a precalibrated refractometer (URC-Ne, Atago, Japan), as previously described [32]. Biochemical variables (pH, microalbuminuria (MA), erythrocytes) were measured by placing a reagent strip (Combur Test, Roche, Spain) in a small portion of urine samples. Next, the strip was placed inside an automatic reflection photometer (Urisys 1100, Roche, Spain) to measure the parameters after a 1 min incubation time.

### 2.8. Statistical Analysis

Data were processed in IBM SPSS 25.0 Statistics for Macintosh (IBM Corp., Armonk, NY, USA) and expressed as the mean ± standard deviation. The normality of the distribution of variables was analyzed by the Shapiro–Wilk test and the homogeneity of variances by the Levene test. The Student’s *t*-test was employed to determine the differences in percentages of change (pre-SC vs. post-SC). A two-way ANOVA (gender effect and SC effect) was used to show any differences in the studied variables. Effect size was calculated by the two-way ANOVA, using partial eta-squared (η2), where 0.01–0.06 was a small effect size, 0.06–0.14 was a moderate effect size, and >0.14 was a large effect size [33]. *p* < 0.05 differences were considered to be statistically significant.

## 3. Results

The results obtained in this study are provided below. A two-way ANOVA was used, as shown in Table 3, Table 4, Table 5 and Table 6. Table 1 presents the results for the variables body weight and hand grip strength. Significant gender differences appeared in all the variables (*p* < 0.001).

Table 4 shows the results obtained in the jump tests. Significant gender differences appeared in all the variables, except for watts, relating to body weight in ABK (*p* < 0.05). Significant post-SC differences were found for ABK (*p* < 0.05).

Table 5 presents the results obtained in the urinary analysis. Significant gender differences were found for specific gravity (*p* < 0.05). For the SC effect, differences appeared for MA (*p* < 0.01).

Table 6 provides the study participants’ hematological parameters. Significant gender differences were found in all the studied variables (*p* < 0.001).

**Figure 1:**
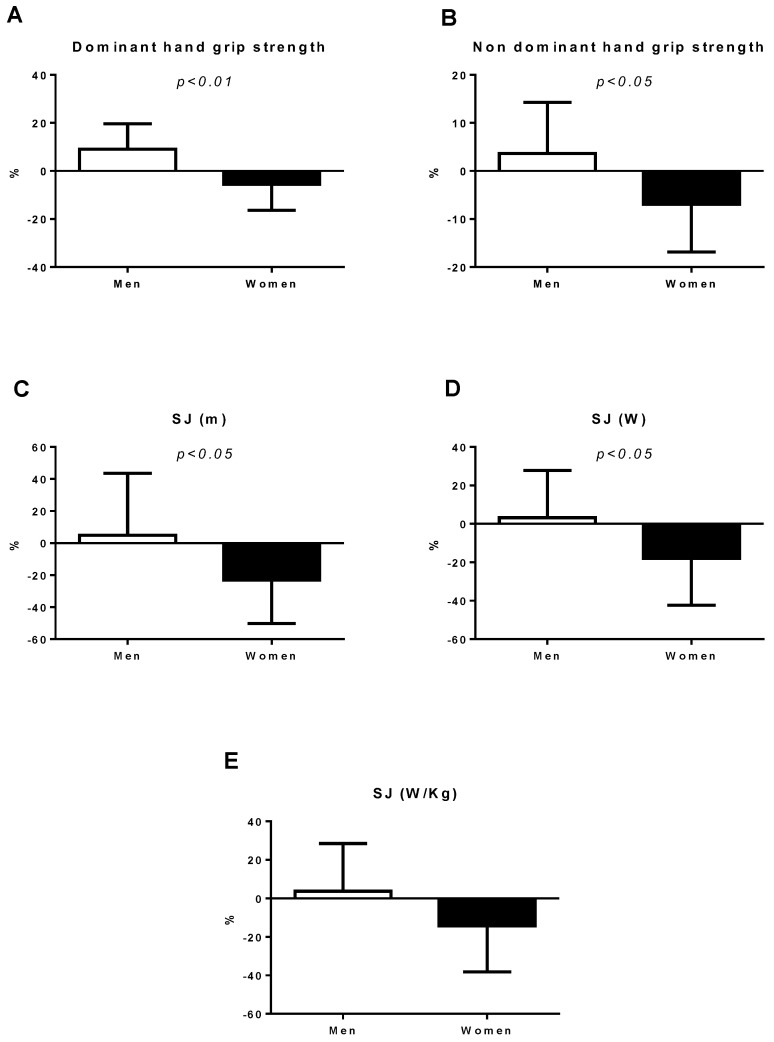
The percentages of change in the hand grip strength and neuromuscular parameters: (**A**) The percentage of change in the dominant hand grip strength; (**B**) the percentage of change in the non-dominant hand grip strength; (**C**) the percentage of change in SJ; (**D**) the percentage of change in the SJ watts; (**E**) the percentage of change in the watts in relation to body weight SJ; SJ: squat jump.

**Figure 2:**
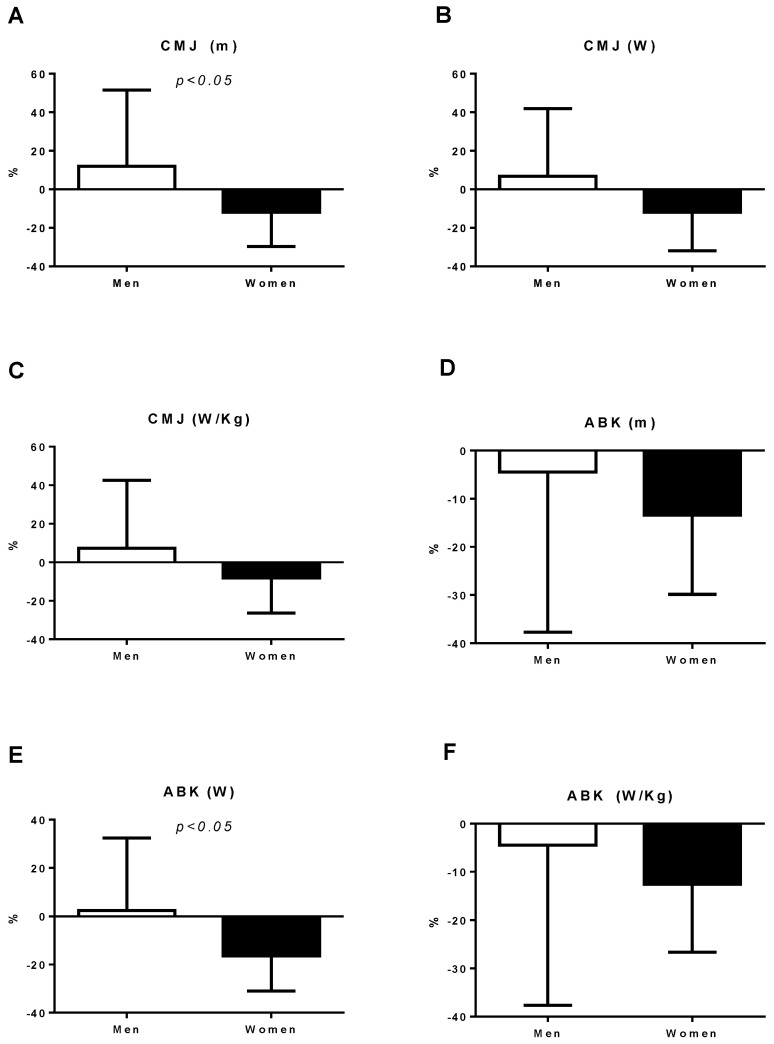
The percentages of change in the neuromuscular parameters (CMJ and ABK): (**A**) The percentage of change in CMJ height; (**B**) the percentage of change in CMJ watts; (**C**) the percentage of change in the watts in relation to body weight CMJ; (**D**) the percentage of change in ABK height; (**E**) the percentage of change in ABK watts; (**F**) the percentage of change in the body weight watts. CMJ: countermovement jump; ABK: Abalakov jump.

Figure 1 and Figure 2 illustrate the percentages of change in the analyzed neuromuscular parameters. Student’s *t*-test was used to determine differences in percentage change (pre-SC vs. post-SC). Data were expressed as the mean ± standard deviation. Significant differences in hand grip strength were found: SJ (height and watts), CMJ (height), and ABK (watts), with more marked changes for men (*p* < 0.05).

## 4. Discussion

The objectives of the present study were to analyze (a) neuromuscular, urinary, and hematological responses after a match and (b) possible gender differences. The SC brought about significant changes in both ABK height and MA in all the study participants. For gender differences, the percentages of change in hand grip strength, SJ (height and watts), CMJ (height), and ABK (height) were higher in men than women. As far as we know, this is the first study to analyze neuromuscular response after padel matches. Although the hematological changes after padel matches have been previously reported [10], the present research work provides information about neuromuscular and urinary changes. The participants’ hematological [34] and urinary [35] parameters fell within the normal ranges.

The hand grip strength evaluation is employed in many sports [36], especially racket sports [37]. The present study found gender differences in both the absolute values (*p* < 0.001) and the post-SC percentage of change (*p* < 0.05). The values obtained herein in men for hand grip strength were similar to those reported for padel by Sánchez-Muñoz et al., [7] in male elite players. Regarding gender differences, previous studies have found higher hand grip strength values generally for men [38] and for tennis [39,40] and badminton players [37], compared to women. The present study found asymmetric hand grip strength regarding padel, similar to other studies [7] and sports [41,42]. Lack of post-SC hand grip strength changes coincides with the results obtained for badminton [43]. Apparently, racket sports report no gender differences in forearm muscle fatigability during intermittent fist submaximal contractions, regardless of muscle strength [44]. This means that the physical stress induced during SC did not affect upper limb capacity to generate strength.

Significant gender differences were noted in the jump tests for all the variables, except the watts, related to body weight for ABK (*p* < 0.05). The percentage of post-SC change was found in men, who performed slightly better in SJ (height and watts), CMJ (height), and ABK (watts) than women (*p* < 0.05). The post-SC differences were significant in ABK height (*p* < 0.05). In general terms, the power recorded in jumps did not change after SC and even slightly improved in men. Jump tests were employed to evaluate neuromuscular and metabolic fatigue [45]. The reported data herein suggest that SC did not cause neuromuscular fatigue in the lower body. These results fall in line with those reported in other racket sports, such as tennis [19,46,47,48,49] or badminton [37,43]. Nevertheless, other authors have obtained contradictory results for the height and mean power in CMJ after playing a tennis match [50]. These findings could be attributed to not only different court surfaces, but also each typical intermittent-type physiological response of these sports, because padel matches involve long breaks that might even prevent neuromuscular fatigue. While playing high-intensity intermittent sports, recovery periods play a key role in limiting fatigue [21]. Moreover, the gender differences found for the jump variables agree with those previously obtained in padel players, regardless of their level [4,51]. The significantly higher height/power values obtained by men in jumps versus women might be due to anthropometric and strength differences between both genders, a circumstance that also appears not only with high-level tennis and badminton players but also with other sport disciplines [52].

Hematological differences appeared in men, obtaining higher values for RBC, hematocrit, and hemoglobin than women (*p* < 0.001). These differences have also been previously reported for padel players [10], swimmers [53], and other sports [54]. Hormone differences and differences in muscle mass and physical condition between genders could influence hematological values [55,56,57].

The urinary values revealed a significant gender difference for specific gravity (*p* < 0.05). The SC effect also led to a difference in MA (*p* < 0.01). Urine specific gravity has been used to measure sportspeople’s hydration status [58,59]. A specific gravity value of >1020 g/mL is used to indicate dehydration [60]. Thus, according to the results reported herein, our participants presented dehydration when they began SC. After the match, men’s dehydration status became worse, but it improved in women. Similar to our study, Silva et al. [58] reported lower values for women than men, while Volpe et al. [61] demonstrated that 13% of student athletes were significantly hypohydrated, with a higher percentage for men hypohydrated (47%) than hypohydrated women (28%). The observed gender differences in specific gravity could be related to women’s anthropometric and game characteristics. Women tend to perform pieces of play more slowly and have a smaller body size and lower metabolic rates, which can all imply less sweating than men [62], which would facilitate better rehydration while playing matches. Regarding increased MA, some studies have observed how this parameter increases after intense physical exercise [63,64]. High-intensity physical exercise can lead to hematuria and proteinuria appearing. These urinary anomalies are related to constricting kidney blood vessels that slow down renal plasma flow during exercise [65]. The increase in MA noted in the present study could be the result of greater glomerular permeability or filtered load or due to a reduction in tubular absorption caused by physical exercise [63,66].

The present study has some limitations. First of all, the match was not real, which could affect the results because players’ expectations and motivations could differ. Secondly, no changes in plasma were included. Thirdly, our small sample size limited the generalization of the obtained results. Fourthly, this study was conducted on an outdoors court, whereas, today, padel is increasingly played indoors. The results could differ on indoor courts because of the distinct temperature and humidity conditions. Finally, game surfaces have been recently modified, which could influence the results obtained in the present research work. Therefore, conducting new studies could be very interesting.

## 5. Conclusions

According to our results, SC did not negatively affect men’s neuromuscular fatigue, unlike women, who were more affected. Regarding urinary and hematological changes, SC affected only MA excretion in both genders.

These differences could be due to both genders’ physiological and anthropometric characteristics and to differences in game actions and the temporal structure while playing padel matches. Further research is necessary to elucidate these facts. 

## Figures and Tables

**Table 1:** Participants’ characteristics.

Parameters	Men (n = 13)	Women (n = 15)	*p*
Age (years)	26.83 ± 6.57	30.07 ± 4.36	0.056
Weight (kg)	75.75 ± 7.88	63.52 ± 4.14	<0.001
Height (m)	1.76 ± 0.03	1.67 ± 0.04	<0.001
Fat (%)	14.40 ± 5.07	20.65 ± 2.64	<0.001
VO_2max_ (ml/kg/min)	57.10 ± 5.37	46.91 ± 4.31	<0.001
Maximum heart rate (bpm)	188.16 ± 10.52	182.16 ± 10.52	0.655
Experience (years)	7.25 ± 2.76	10.87 ± 3.66	0.009
Weekly training (h)	21.42 ± 3.37	22.47 ± 4.01	0.973

VO_2max_: maximal oxygen consumption.

**Table 2:** SC characteristics.

Parameters		Men (n = 13)	Women (n = 15)	*p*
Side played (%)	Forehand	38.5	40.0	0.933
	Backhand	61.5	60.0	
Mean heart rate (bpm)		152.46 ± 9.96	144.75 ± 18.56	0.179
Maximum heart rate (bpm)		175.26 ± 9.27	173.25 ± 18.23	0.712
Total time (min)		75.81 ± 18.34	70.32 ± 14.04	0.318
Real time (min)		26.78 ± 8.49	25.31 ± 7.43	0.730
Rest time (min)		50.13 ± 9.21	47.65 ± 8.14	0.415
Relative humidity (%)		42.17 ± 7.69	46.02 ± 3.12	0.109
Temperature (°C)		25.93 ± 5.58	24.33 ± 9.24	0.662
Water intake (mL)		795.21 ± 254.12	713.41 ± 301.81	0.211

**Table 3:** The sample’s body weight and hand grip strength.

Parameters	Time	Men (n = 13) (M ± SD)	Women (n = 15) (M ± SD)	Gender Effect	η2	SC Effect	η2	Gender × SC	η2
Weight (kg)	Pre	75.75 ± 7.88	63.52 ± 4.14	<0.001	0.623	0.789	0.001	0.482	0.010
	Post	76.29 ± 8.06	62.12 ± 5.30						
Dominant hand grip strength (kg)	Pre	50.50 ± 9.59	34.46 ± 4.94	<0.001	0.663	0.507	0.009	0.083	0.058
	Post	55.24 ± 9.20	32.32 ± 4.27						
Non-dominant hand grip strength (kg)	Pre	44.42 ± 7.60	27.95 ± 2.62	<0.001	0.721	0.842	0.001	0.174	0.036
	Post	47.00 ± 8.72	26.03 ± 3.83						

SC: simulated padel competition; η2: eta-squared.

**Table 4:** Results obtained in the jump tests for both groups.

Parameters	Time	Men (n = 13) (M ± SD)	Women (n = 15) (M ± SD)	Gender Effect	η2	SC Effect	η2	Gender × SC	η2
SJ (m)	Pre	0.270 ± 0.09	0.214 ± 0.04	<0.001	0.222	0.173	0.036	0.260	0.025
	Post	0.262 ± 0.09	0.164 ± 0.062						
SJ (W)	Pre	2835.08 ± 675.04	2037.98 ± 476.46	<0.001	0.508	0.233	0.028	0.121	0.047
	Post	2906.31 ± 509.93	1638.66 ± 462.43						
SJ (W/kg)	Pre	36.27 ± 8.09	33.69 ± 7.80	0.011	0.121	0.210	0.031	0.128	0.035
	Post	36.45 ± 4.87	28.44 ± 8.07						
CMJ (m)	Pre	0.319 ± 0.08	0.243 ± 0.05	<0.001	0.276	0.859	0.001	0.240	0.027
	Post	0.343 ± 0.141	0.210 ± 0.04						
CMJ (W)	Pre	3135.00 ± 713.17	2213.06 ± 480.28	<0.001	0.544	0.489	0.009	0.180	0.035
	Post	3230.06 ± 479.42	1918.52 ± 436.69						
CMJ (W/kg)	Pre	39.98 ± 8.95	36.64 ± 7.99	0.007	0.135	0.424	0.013	0.284	0.022
	Post	40.51 ± 3.72	33.02 ± 6.60						
ABK (m)	Pre	0.376 ± 0.09	0.289 ± 0.05	<0.001	0.296	0.036	0.084	0.926	0.000
	Post	0.336 ± 0.07	0.246 ± 0.04						
ABK (W)	Pre	3437.58 ± 864.36	2539.93 ± 486.07	<0.001	0.480	0.136	0.043	0.235	0.027
	Post	3387.61 ± 517.14	2109.06 ± 442.69						
ABK (W/kg)	Pre	43.79 ± 10.91	42.22 ± 8.94	0.080	0.059	0.103	0.051	0.294	0.022
	Post	42.49 ± 4.31	36.32 ± 6.61						

SC: simulated padel competition; SJ: squat jump; CMJ: countermovement jump; ABK: Abalakov jump; η2: eta-squared.

**Table 5:** Urinary parameters before and after CS in both groups.

Parameters	Time	Men (n = 13) (M ± SD)	Women (n = 15) (M ± SD)	Gender Effect	η2	SC Effect	η2	Gender × SC	η2
Specific gravity (g/mL)	Pre	1.024 ± 0.004	1.020 ± 0.005	0.015	0.110	0.576	0.006	0.981	0.000
	Post	1.023 ± 0.005	1.019 ± 0.006						
pH	Pre	5.83 ± 0.88	6.50 ± 0.73	0.268	0.024	0.332	0.018	0.099	0.052
	Post	6.00 ± 0.84	5.86 ± 1.02						
MA (g/L)	Pre	47.25 ± 18.81	46.52 ± 33.67	0.609	0.005	0.001	0.210	0.599	0.005
	Post	207.51 ± 147.11	260.47 ± 328.64						
RBC (cells/µL)	Pre	0.833 ± 2.88	0.661 ± 0.25	0.242	0.027	0.136	0.043	0.211	0.031
	Post	3.076 ± 4.80	25.332 ± 63.76						

SC: simulated padel competition; η2: eta-squared; MA: microalbuminuria; RBC: red blood cells.

**Table 6:** The participants’ hematological parameters.

Parameters	Time	Men (n = 13) (M ± SD)	Women (n = 15) (M ± SD)	Gender Effect	η2	SC Effect	η2	Gender × SC	η2
RBC (x10^12^/L)	Pre	4.99 ± 0.42	4.23 ± 0.21	<0.001	0.637	0.803	0.001	0.791	0.001
	Post	4.99 ± 0.35	4.19 ± 0.20						
Hb (g/L)	Pre	154.16 ± 10.92	133.06 ± 6.89	<0.001	0.656	0.555	0.007	0.758	0.002
	Post	153.53 ± 8.46	131.06 ± 6.31						
Hct (L/L)	Pre	0.44 ± 0.03	0.38 ± 0.02	<0.001	0.622	0.602	0.005	0.844	0.001
	Post	0.447 ± 0.02	0.385 ± 0.01						

SC: simulated padel competition; η2: eta-squared; RBC: red blood cells; Hb: hemoglobin; Hct: hematocrit.

## Data Availability

The data presented in this study are available on request from the corresponding author. The data are not publicly available due to privacy.
